# Mr. Coley's Case of Urinous Vomiting

**Published:** 1813-12

**Authors:** J. M. Coley

**Affiliations:** Bridgnorth


*To the Editor of the Medical and Physical Journal.*

SIR,

HAVING observed in your Journal for July last, that there has existed a difference of opinion between Dr. Yeates and several other medical persons in Bedford, respecting a case of Ischuria, in which it has been asserted that the patient vomited her urine; and Dr. Y. having respectfully solicited communications from other practitioners on the subject, I beg to present your readers with the following case, which occurred in my practice, and which, I trust, will be considered as an additional proof of the occasional occurrence of urinous vomiting.

On the beginning of June, 1808, Jane Barret, ætat. 21, mother of one child, in going down some stairs fell between two barrels, in consequence of which two of her ribs were fractured near their angles, the spine and hip were much injured, and she remained lame on one side of the body until her death. When the accident happened she resided in Dudley, and became the patient of Mr. Cartwright, of that place. At the end of 20 or 30 hours, Mr. C. found it requisite to introduce the catheter, for the purpose of evacuating the bladder, and of relieving some violent spasmodic pains. While she remained under his care, Mr. C. appeared to have treated the disease with judgment and humanity; and, besides the application of other remedies, he was particularly diligent in the use of the catheter. On July 21, she was removed into this neighbourhood, when I was requested to see her. Owing probably to the tardy arrangements of her friends, the catheter had not then been introduced during two days and nights, and she had discharged no urine. I drew off 10 ounces, and found on enquiry that, since the operation had become needful, this quantity had never been exceeded.

The catheter was employed sometimes once, sometimes twice, daily, until August 5, when it appeared that she could, with much pain and difficulty, evacuate the bladder spontaneously about once in 20 or 30 hours. During that period various medicines were administered with the view of promoting a secretion of urine; for there was a suppression as well as a retention of that fluid.

She was suddenly attacked, on August 1, with a violent pain in the stomach, which required the exhibition of twelve grains of opium to allay it. This returned afterwards on alternate days, and always terminated with sickness, and a vomiting of a fluid, possessing all the properties of healthy urine, with a disagreeable excess of uric acid. This urinous fluid was repeatedly discharged in the presence of myself and several other persons, and was minutely examined. It was observed, that on the days when this pain and vomiting occurred, no urine was discharged from the bladder; but on the intermediate days it was secreted and evacuated in the usual manner.

She remained in this state till October 21, when a troublesome constipation in the bowels commenced, and continued for ten days. This new symptom was probably owing to a tense tumor of considerable magnitude, which had then occupied the region of the kidnies, and was circumscribed, like an encysted dropsy. No fluctuation could be perceived in it, but it appeared to have arisen from a preternatural distention of one or both of the renal pelves, or from some other organic derangement in the kidnies. The pain in the stomach now ceased spontaneously, and the vomiting was attended with hiccough, and with urinous perspiration, which was particularly profuse on the forehead. No urine was discharged from the bladder; and after the bowels had been evacuated, the tumor rather subsided. The pulse continued at 90, the appetite was good, and she had not experienced any emaciation.

In this condition she was removed to the Shrewsbury Infirmary, where she soon afterwards died. I have never heard whether the body was opened after death.

---

I take the liberty of communicating the successful treatment of a case of pulmonary consumption by *Carbonate of Barytes*. The patient, a female, about 20 years of age, had had taken the remedies usually prescribed in such cases, without deriving any advantage; and, having rapidly declined into an advanced stage of the disease, was considered to be beyond the possibility of recovery. She had been confined to bed about three months, expectorated a pint of purulent matter daily, was nearly exhausted by hectic fever, and her respiration was most distressing. While she was in this condition, I began the exhibition of Carbonate of Barytes on August 4, 1813, in the dose of one-sixth of a grain, made into pills with conserve; and on the 13th had gradually increased it to 20 grains, twice daily. At that time a slight sickness commenced, on which account the medicine was discontinued; and in a few days afterwards an eruption (lichen simplex) appeared over the whole body. When this event happened, the symptoms of pulmonary disease began to diminish; and after it had continued three weeks, they entirely subsided, leaving the patient in good health. Having had no previous experience with the Carbonate of Barytes, which has, I believe, been considered to possess very deleterious properties, I cannot say whether the eruption on the skin might have been produced by its influence or not; but may perhaps be allowed to recommend a farther trial of it in hopeless cases of pulmonary phthisis, where the experiment might be admissible, and the practitioner might have time and opportunity to notice its operation.

*Bridgnorth,* I am, Sir, yours, &c.

*Oct.* 27, 1813. J. M. COLEY.

