# Corrigenda

**Published:** 1813-12

**Authors:** 


					CORRIGENDA.
For Dr. Yeates, p. 465 read Dr. Yeats.

				

